# Development of oseltamivir and zanamivir resistance in influenza A(H1N1)pdm09 virus, Denmark, 2014

**DOI:** 10.2807/1560-7917.ES.2017.22.3.30445

**Published:** 2017-01-19

**Authors:** Ramona Trebbien, Svend Stenvang Pedersen, Kristine Vorborg, Kristina Træholt Franck, Thea Kølsen Fischer

**Affiliations:** 1 National Influenza Center, Virological Surveillance and Research, Department of Microbiological Diagnostics and Virology, Statens Serum Institut, Denmark; 2 Department of Infectious Diseases, Odense University hospital, Odense, Denmark; 3 Department of Microbiological Diagnostics and Virology, Statens Serum Institut, Denmark; 4 Department of Clinical Microbiology, Copenhagen University Hospital, Herlev, Denmark

## Abstract

Antiviral treatment of immunocompromised patients with prolonged influenza virus infection can lead to multidrug resistance. This study reveals the selection of antiviral resistance mutations in influenza A(H1N1)pdm09 virus in an immunocompromised patient during a 6-month period. The patient was treated with two courses of oseltamivir (5 days and 2 months, respectively), with the first course starting at symptom onset, and subsequently zanamivir (2 months and 10 days, respectively). Respiratory samples were investigated by Sanger and next generation sequencing (NGS) and, for NGS data, low-frequency-variant-detection analysis was performed. Neuraminidase-inhibition tests were conducted for samples isolated in Madin-Darby canine kidney cells. In a sample collected 15 days after the end of the first treatment with oseltamivir (Day 20 post-symptom onset), oseltamivir resistance was detected (mutation H275Y with 60.3% frequency by NGS). Day 149 when the patient had almost completed the second zanamivir treatment, mixes of the following resistance mutations were detected; H275Y(65.1%), I223R(9.2%), and E119G(89.6%), accompanied by additional mutations, showing a more complex viral population in the long-term treated patient. Two samples obtained on Day 151 from bronchoalveolar lavage (BAL) and nasopharyngeal swab, respectively, showed different mutation profiles, with a higher frequency of antiviral resistance mutations in BAL. The results emphasise the importance of timely antiviral resistance testing both for treatment of individual patients as well as for preventive measures to control the development and transmission of antiviral resistant viruses.

## Introduction

Influenza virus is the cause of annual seasonal epidemics worldwide, and leads to high morbidity in the population. Severe disease and deadly outcome due to influenza virus are recognised in the defined risk groups, in particular elderly persons > 65 years of age and immunocompromised patients. Influenza is normally an acute self-limiting disease with a duration of 5 to 7 days, however, in immunocompromised patients prolonged infections lasting several months have been reported [1-4]. Prevention of severe influenza disease is mainly based on immunisations with split vaccines which are produced annually to accommodate the changing antigenicity of seasonal epidemic viruses [5,6]. The effect of vaccination in immunocompromised patients is questionable and this is why other modes of prevention and/or treatment often are considered for this risk group [7-12]. For treatment of influenza viruses only a few antiviral drugs are available; the neuraminidase (NA) inhibitors and the matrix-2 (M2)-ion channel inhibitors. The current circulating epidemic influenza viruses harbour natural resistance towards the M2-ion channel inhibitors therefore these are not an option for treatment [13-15]. The NA inhibitors bind to the NA surface protein and prevent it from facilitating the release of new virus particles from an infected cell [16]. In Denmark, two different NA inhibitors are approved and available for treatment of influenza: oseltamivir (Tamiflu) and zanamivir (Relenza). Oseltamivir is the drug of choice for treatment due to its easy oral administration whereas zanamivir (intravenous or inhalation) is often used when the effect of oseltamivir is limited, e.g. in case of development of resistance. In the NA gene of the H1N1 viruses a range of amino acid mutations are recognised to confer reduced inhibition by NA inhibitors [16-18]. Among these, two well characterised mutations are the H275Y mutation which results in viruses with highly reduced inhibition by oseltamivir and the I223R mutation which results in reduced inhibition by both oseltamivir and zanamivir [16,17].

Antiviral treatment of immunocompromised patients with prolonged influenza virus infection can lead to multidrug-resistant influenza quasispecies in the same patient [1]. We describe how the emergence of such virus variants poses challenges in the combat of a severe influenza infection in a Danish patient treated with antivirals. The patient had sustained shedding of influenza A(H1N1)pdm09 virus for 6 months and was treated with oseltamivir and subsequently zanamivir. Antiviral resistance mutation profiles were evaluated using conventional Sanger sequencing and next generation sequencing (NGS).

## Methods

### Case and samples 

The immunocompromised patient had chronic lymphocytic leukaemia (CLL) and was aged between fifty and sixty years. Influenza-vaccination status was unknown. Respiratory samples were obtained at frequent intervals during the course of infection. These included nasopharyngeal swabs, bronchoalveolar lavage (BAL), and expectorates, which were used for diagnostic of influenza virus and detection of antiviral resistance. The first sample was collected at the onset of respiratory symptoms. Since it tested positive for influenza A(H1N1)pdm09, oseltamivir treatment was immediately started. Approximately 3 months later, a first sample for antiviral resistance testing was submitted to the National Influenza Center, Denmark. Subsequent samples were then also investigated, as well as those collected before this time point, which were retrospectively analysed. The overall treatment consisted of two courses of oral oseltamivir, one course of inhalation therapy with zanamivir, and a compassionate-use programme with intra venous (i.v.) zanamivir (Figure).

**Figure:**
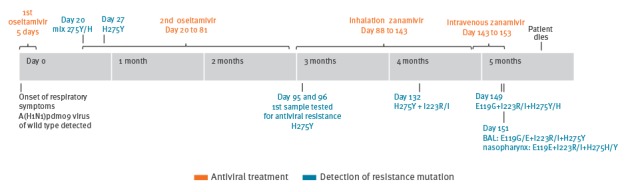
Time line of the treatment course of an immunocompromised patient with influenza with oseltamivir and zanamivir and of development of antiviral resistance mutations, Denmark, 2014

BAL: bronchoalveolar lavage.

### Virus detection

Total nucleic acid was extracted using 200 µL of sample material and the MagNA Pure LC Total Nucleic Acid Isolation Kit on the MagNapure 96/32 (Roche).

A multiplex real-time reverse transcription-PCR (RT-PCR) detecting the influenza A matrix (M) gene as well as H1N1pdm09 NA gene was performed using the MX3005P Stratagene platform according to the protocol designed by the National Influenza Center Denmark (Statens Serum Institut, Copenhagen, Denmark). The cycle threshold (Ct) values for the M-gene were used as a relative measure of viral load.

### Virus isolation

Isolation of influenza virus was performed in duplicates in Madin-Darby canine kidney (MDCK) cells using standard methods [19]. Because of challenges caused by poor constitution/lack of sample material and difficulties in cultivating the virus in ca 50% of samples, it was not possible to report viral load in PFU/mL or TCID50.

Due to loss of the zanamivir antiviral resistance mutations during cell-propagation, experimentation with addition of zanamivir and oseltamivir in different concentrations and combinations was performed in an attempt to rescue the mutated virus. Virus growth medium was supplemented with zanamivir and oseltamivir at the following concentrations: 1 µM, 0.1 µM, 0.01 µM, 0.001 µM. The two drugs were mixed or added alone to the virus growth medium in the different concentrations.

### Detection of the H275Y mutation using allele-specific real-time reverse transcription-PCR

For rapid initial screening of the oseltamivir resistance conferring single nt polymorphism (SNP) mutation H275Y, an allele specific real-time RT-PCR was applied to the samples, following a protocol designed by the National Influenza Center Denmark (Statens Serum Institut, Copenhagen, Denmark).

### Sequencing of the neuraminidase gene and minority variant analysis

Full-length Sanger sequencing of the NA gene was performed by RT-PCR using in-house primers and Big Dye chemistry on an ABI3500 capillary sequencer (Applied Biosystems) and was analysed using Bionumerics (Applied Maths, Belgium) and Molecular Evolutionary Genetics Analysis (MEGA)6 software.

NGS was performed using NexteraXT DNA sample preparation kit (Illumina) and subsequent sequencing on the MiSeq (Illumina).

Minority variant analysis was performed on NGS data using CLC Genomics Workbench version 8 (Qiagen, Germany).

### Neuraminidase inhibition assays

Oseltamivir and zanamivir inhibition of the NA activity of virus isolates was assessed using the NA-Fluor Influenza Neuraminidase Assay Kit from Applied Biosystems (Life technologies). The reference virus A/California/07/2009(H1N1pdm09) was used as wild type control. The mean 50% inhibitory concentration (IC) was calculated according to the kit protocol and as the fold-change to the wild-type control virus. World Health Organization (WHO) criteria for determination of inhibition by oseltamivir and zanamivir were used to evaluate the level of antiviral resistance [20]. Normal inhibition (NI) is < 10 IC50 fold change compared with wild-type virus, reduced inhibition (RI) 10–100, and highly reduced (HR) > 100.

## Results

### Genotypic antiviral resistance testing results

The patient was treated with oseltamivir immediately after diagnosis. The sample from day 0, the same day the first oseltamivir treatment was initiated, was retrospectively analysed for antiviral resistance mutations. At this point there was no antiviral resistance mutations recognised (Table 1).

**Table 1:** Amino acid substitutions in the influenza A(H1N1)pdm09 virus neuraminidase, reported to be involved in antiviral resistance [16], which were found in an immunocompromised patient treated with oseltamivir and zanamivir, Denmark 2014

Sample number: cells for virus culture and antiviral added if any	Day	Origin of the sample	M-gene Ct-value	Reads NGS	Sequencing method	Amino acid position and type in the consensus sequence of reference virus A/California/07/2009; for comparison to the sequence of the virus infecting the patient								
						117^a^	118^b^	119^b^	136^a^	199^b^	223^b^	247^a^	275^b^	402^b^
						I	R	E	Q	D	I	S	H	Y
1	0	Nasopha.	29.55	1,525,617	NGS (%)	*	*	*	*	*	*	*	*	*
					S	*	*	*	*	*	*	*	*	*
2	20	Nasopha.	27.47	914,914	NGS (%)	M (1.04)	*	*	*	*	R (3.4)	*	Y (60.3)	*
					S	*	*	*	*	*	*	*	Y/H	*
3	27	Nasopha.	31.5	4,483	NGS (%)	*	*	*	*	*	*	*	Y (> 99)	*
					S	n.d.	n.d.	n.d.	n.d.	n.d.	n.d.	n.d.	n.d.	n.d.
4	95	BAL	25.88	n.d.	NGS (%)	n.d.	n.d.	n.d.	n.d.	n.d.	n.d.	n.d.	n.d.	n.d.
					S	*	*	*	*	*	*	*	Y	*
4: 2MDCK Z		Cell culture	34.35	1,216,299	NGS (%)	*	*	G (24.3)	*	*	*	N (72.4)	Y (> 99)	*
					S	*	*	E/G	*	*	*	S/N	Y	n.d.
4: 2MDCK Z/O		Cell culture	34.53	1,566,074	NGS (%)	*	*	G (30.4)	*	G (1.6)	*	N (42.6)	Y (> 99)	*
					S	*	*	E/G	*	*	*	S/N	Y	*
5	96	BAL	26.53	n.d.	NGS (%)	n.d.	n.d.	n.d.	n.d.	n.d.	n.d.	n.d.	n.d.	n.d.
					S	*	*	*	*	*	*	S/N	Y	*
5: 3MDCK		Cell culture	16.82	1,458,499	NGS (%)	*	*	*	*	*	*	*	Y (> 99)	N (1.47)
					S	*	*	*	*	*	*	*	Y	*
5: 4MDCK Z		Cell culture	22.96	1,233,121	NGS (%)	*	*	*	*	*	*	*	Y (> 99)	*
					S	*	*	*	*	*	*	n.d.	Y	*
5: 4MDCK Z/O		Cell culture	23.72	n.d.	NGS (%)	n.d.	n.d.	n.d.	n.d.	n.d.	n.d.	n.d.	n.d.	n.d.
					S	*	*	*	*	*	*	*	Y	*
6	132	Expectorate	28.83	1,132,787	NGS (%)	*	*	*	*	*	R (53.4)	R (1.1)	Y (> 99)	*
					S	*	*	*	*	*	I/R	*	Y	*
6: 2MDCK		Cell culture	18.73	1,012,035	NGS (%)	*	*	*	*	*	*	*	Y (> 99)	*
					S	*	*	*	*	*	*	*	Y	*
6: 2MDCK Z		Cell culture	32.01	2,955,354	NGS (%)	*	*	*	*	*	R (75.3)	*	Y (> 99)	*
					S	*	*	*	*	*	I/R	*	Y	*
6: 2MDCK Z/O		Cell culture	34.13	641,179	NGS (%)	*	*	*	*	*	R (49.3)	I (1.9)	Y (> 99)	*
					S	*	*	*	*	*	I/R	*	Y	*
6: 3MDCK		Cell culture	18.71	1,255,140	NGS (%)	*	*	*	*	*	*	*	Y (> 99)	*
					S	*	*	*	*	*	*	*	Y	*
7	149	Expectorate	32.61	1,413,804	NGS (%)	L (7.04)/M (9.3)	*	G (89.6)	*	*	R (9.2)	N (6.7)	Y (65.1)	*
					S	*	*	G/E	*	*	*	*	H/Y	*
8	151	BAL	36.99	3,036,466	NGS (%)	*	*	G (35.9)	*	*	R (51.8)	*	Y (88.2)	*
					S	*	*	E/G	*	*	I/R	*	Y	*
8: 2MDCK		Cell culture	17.93	1,823,988	NGS (%)	*	*	*	*	*	*	*	Y (> 99)	*
					S	*	*	*	*	*	*	*	Y	*
8: 3MDCK		Cell culture	15.94	1,274,826	NGS (%)	*	*	*	*	*	*	*	Y (> 99)	*
					S	*	*	*	*	*	*	*	Y	*
9	151	Nasopha.	36.56	1,699,587	NGS (%)	*	M (1.1)	G (7.3)	K (2.5)	*	R (34.2)	N (6.2)	Y (74.9)	*
					S	*	*	*	*	*	I/R	*	H/Y	*

AA: amino acid; BAL: bronchoalveolar lavage; Ct-value: cycle threshold value obtained by real-time reverse transcription-PCR; M-gene: matrix gene; MDCK: Madin-Darby canine kidney cells; nasopha.: nasopharyngeal swab; n.d.: no data; NGS: next generation sequencing; S: Sanger sequencing; z: virus isolate cultivated with 1µM zanamivir; z/o: virus isolate cultivated with 1µM zanamivir and 0.1µM oseltamivir.

The samples are ordered and numbered in the chronological order of collection. When the virus was isolated in culture from a sample, the types of cells and the number of passages are indicated next to the sample number. The Table also depicts the type of sample material, which was initially collected. NGS and S-sequencing of samples or the respective virus isolates were performed to infer the AA sequence. When more than one AA type was inferred at a given sequence position by NGS, the frequency of each AA identified is provided (in the rows: NGS (%)). The * indicates that the AA is identical to the reference virus A/California/07/2009(H1N1pdm09) consensus sequence.

^a^ Non-active site of the neuraminidase enzyme.

^b^ Active site of the neuraminidase enzyme.

Despite the first oseltamivir treatment lasting 5 days, the patient continued to have symptoms and influenza A(H1N1)pdm09 positive samples. As a result, a second oseltamivir treatment was initiated at 20 days post-symptom onset (Day 20). Samples collected 15 days after termination of the 1^st^ oseltamivir treatment (Day 20) and 7 days after initiation of the 2^nd^ oseltamivir treatment (Day 27), were retrospectively investigated as well. Both contained virus with the H275Y mutation at a frequency of 60.3% (day 20) and > 99% (day 27). Day 96, one week after initiation of inhalation therapy with zanamivir and three months after initiation of symptoms including two courses of treatment with oseltamivir another sample was collected and submitted for antiviral resistance testing to the National Influenza Center. The sample contained influenza A(H1N1)pdm09 virus with the H275Y mutation and Sanger sequencing revealed an additional S247N mutation(Figure, Table 1). Day 132, one and a half month after initiation of inhalation therapy with zanamivir a sample was investigated for further development of antiviral resistance mutations. At this time point the H275Y mutation was still recognised, however, a mixed population with the I223R/I mutation was also observed by Sanger sequencing. The I223R substitution was later confirmed by NGS with a frequency of 53.4% (Figure, Table 1).

As no clinical improvement of the patient was obtained, i.v. zanamivir treatment was carried out for 10 days. Samples from Day 149 and 151, six and eight days after initiation of i.v. zanamivir treatment, respectively, revealed a mixed population of virus with wild type and resistant-conferring residues at position 275 (H275Y/H) as well as at position 223 (I223R/I) using Sanger sequencing (Figure, Table 1). By NGS a more differentiated viral population was observed involving a range of mutations (Table 1 and 2). Interestingly, a discrepancy was discovered between two samples collected on day 151. In a sample obtained as BAL there was a higher frequency of the major resistance-inducing mutations (E119G: 35.9%, I223R: 51.8%, and H275Y: 88.2%) compared with a sample obtained as nasopharyngeal swab (E119G: 7.3%, I223R: 34.2% and H275Y:74.9%). The nasopharyngeal swab on the other hand showed three additional mutations related to antiviral resistance, however, at a low frequency (R118M: 1.1%, Q136K: 2.5%, and S247N: 6.2%). 

**Table 2:** Influenza A(H1N1)pdm09 virus neuraminidase amino acid substitutions proposed to be involved in viral fitness and related to antiviral resistance and cell adaptation, which were found in samples from an immunocompromised patient treated with oseltamivir and zanamivir, Denmark 2014

Sample	Day	Material	Reads NGS	Sequencing method	Amino acid position and type in the consensus sequence of reference virus A/California/07/2009; for comparison to the sequence of the virus infecting the patient											
					83	86	147	149	173	232	241	248	313	314	416	438
					V	A	G	I	R	A	I	D	Q	I	D	T
1	0	Nasopha.	1,525,617	NGS (%)	*	*	*	*	*	*	*	*	*	*	*	*
				S	*	*	*	*	*	*	*	*	*	*	*	*
2	20	Nasopha.	914,914	NGS (%)	*	*	*	*	*	*	*	*	*	*	N (20.4)	*
				S	*	*	*	*	*	*	*	*	*	*	*	*
3	27	Nasopha.	4,483	NGS (%)	*	*	*	*	*	*	*	*	*	*	*	*
				S	n.d.	n.d.	n.d.	n.d.	n.d.	n.d.	n.d.	n.d.	n.d.	n.d.	n.d.	n.d.
4	95	BAL	n.d	NGS (%)	n.d.	n.d.	n.d.	n.d.	n.d.	n.d.	n.d.	n.d.	n.d.	n.d.	n.d.	n.d.
				S	*	*	*	*	*	*	*	*	*	*	*	*
4: 2MDCK Z		Cell culture	1,216,299	NGS (%)	*	*	R (2)	*	*	*	V (7.7)	N (1.6)	*	*	*	*
				S	*	*	*	*	*	*	*	*	*	*	n.d.	n.d.
4: 2MDCK Z/O		Cell culture	1,566,074	NGS (%)	*	*	*	*	*	*	V (4.5)	N (1.9)	*	T (3.2)	*	A (4.5)
				S	*	*	*	*	*	*	*	*	*	*	*	*
5	96	BAL	n.d.	NGS (%)	n.d.	n.d.	n.d.	n.d.	n.d.	n.d.	n.d.	n.d.	n.d.	n.d.	n.d.	n.d.
				S	*	*	*	*	*	*	*	D/(N)	*	*	*	*
5: 3MDCK		Cell culture	1,458,499	NGS (%)	*	*	*	*	*	*	V (10.6)	*	*	*	*	*
				S	*	*	*	*	*	*	*	*	*	*	*	*
5: 4MDCK Z		Cell culture	1,233,121	NGS (%)	*	*	*	*	*	*	V (4.8)	*	*	*	*	*
				S	*	*	*	*	*	*	*	*	*	*	*	*
5: 4MDCK Z/O		Cell culture	n.d.	NGS (%)	n.d.	n.d.	n.d.	n.d.	n.d.	n.d.	n.d.	n.d.	n.d.	n.d.	n.d.	n.d.
				S	*	*	*	*	*	*	*	*	*	*	*	*
6	132	Expectorate	1,132,787	NGS (%)	I (44.9)	*	R (33.4)	T (6.6)	*	*	V (5.1)	*	K (3.5)	*	N (4.1)	A (1.8)
				S	V/I	*	G/(R)	*	*	*	*	*	*	*	*	*
6: 2MDCK		Cell culture	1,012,035	NGS (%)	*	T (71)	*	T (23)	K (30)	*	V (3.8)	*	K (70)	*	*	*
				S	*	T	*	*	R/K	*	*	*	K	*	*	*
6: 2MDCK Z		Cell culture	2,955,354	NGS (%)	I (14.8)	*	R (15.4)	T (4.1)	*	*	V (1.7)	*	K (4.1)	*	N (2.7)	*
				S	V/(I)	*	*	*	*	*	*	*	*	*	*	*
6: 2MDCK Z/O		Cell culture	641,179	NGS (%)	I (41.6)	*	R (40)	T (6.9)	*	*	V (4)	*	K (2.6)	*	N (1.1)	*
				S	V/I	*	G/R	*	*	*	*	*	*	*	*	*
6: 3MDCK		Cell culture	1,255,140	NGS (%)	*	T (80)	*	T (16)	K (21)	*	V (3.8)	*	K (79)	*	*	*
				S	*	T	*	*	R/K	*	*	*	K	*	*	*
7	149	Expectorate	1,413,804	NGS (%)	I (4.6)	*	R (2)	*	*	V (21.8)	V(3)	N (46)	R (2.8)	T (17.8)	*	A (56.9)
				S	*	*	*	*	*	*	*	D/N	*	*	*	T/A
8	151	BAL	3,036,466	NGS (%)	I (6.1)	*	R (21.3)	*	*	V (14)	V (8.1)	N (9.2)	*	T (5.6)	*	A (3)
				S	*	*	*	*	*	*	*	*	*	*	*	*
8: 2MDCK		Cell culture	1,823,988	NGS (%)	*	T (62)	*	T (32)	K (40)	*	V (3.1)	*	K (60)	*	*	*
				S	*	T	*	I/(T)	R/K	*	*	*	Q/K	*	*	*
8: 3MDCK		Cell culture	1,274,826	NGS (%)	*	T (74)	*	T (22)	K (29)	*	V (5.3)	*	K (71)	*	*	*
				S	*	T	*	*	R/K	*	*	*	K	*	*	*
9	151	Nasopha.	1,699,587	NGS (%)	*	*	*	*	*	V (15.3)	V (5.4)	N (44.9)	R (9.9)	T (8.8)	*	A (2.5)
				S	*	*	*	*	*	*	*	D/N	*	*	*	*
Proposed function^a^					f	c	f	u	c	f	u	f	c	f	u	f

AA: amino acid; BAL: bronchoalveolar lavage; Ct-value: cycle threshold value obtained by real-time reverse transcription-PCR; nasopha.: nasopharyngeal swab; n.d.: no data; MDCK: Madin-Darby canine kidney cells; NGS: next generation sequencing; S: Sanger sequencing; z: virus isolate cultivated with 1µM zanamivir; z/o: virus isolate cultivated with 1µM zanamivir and 0.1µM oseltamivir.

The samples are ordered and numbered in the chronological order of collection. When the virus was isolated in culture from a sample, the type of cells and the number of passages are indicated next to the sample number. The Table also depicts the type sample material, which was initially collected. NGS and Sanger sequencing of samples or the respective virus isolates were performed to infer the AA sequence. When more than one AA type was found at a given sequence position by NGS, the frequency at which each AA identified is provided (in the rows: NGS (%)). The * indicates that the AA is identical to the reference virus A/California/07/2009(H1N1pdm09) consensus sequence.

^a^ f: fitness related to antiviral resistance; c: cell adaptation due to cultivation; u: unknown.

### Genotypic antiviral resistance testing of cell propagated samples 

It was possible to propagate virus from four of the samples in MDCK cells. During propagation it was observed by sequencing that the antiviral resistance mutation I223R found in the original sample materials was lost after cell propagation, while new mutations indicative of cell adaptation occurred at positions A86T, R173K, and Q313K (Table 1 and 2). In an attempt to preserve the antiviral resistance mutations, the growth medium was supplemented with zanamivir alone or zanamivir and oseltamivir. For two virus isolates propagated with antivirals in the growth medium it was possible to rescue viruses with the I223R mutation (Table 1).

### Phenotypic antiviral resistance testing results

Due to a low amount of sample material it was not possible to perform NA inhibition tests on any of the samples directly. Three virus isolates carrying only the H275Y mutation had mean IC50s against oseltamivir, which were ca 500–900 fold higher than the wild type H275 strain A/California/07/2009(H1N1pdm09) virus, thereby showing highly reduced inhibition (Table 3). Against zanamivir there was normal inhibition of the virus isolates carrying the H275Y only. Unfortunately the virus isolates carrying both the I223R/I and H275Y mutations did not display NA activity and the phenotypic NA inhibition by oseltamivir and zanamivir could not be determined for these isolates.

**Table 3:** Results from neuraminidase inhibition assay of virus isolates, Denmark, 2014

**Virus isolate**	**Amino acid substitution**	**Mean IC50 (nM) for oseltamivir**	**Fold change to wild type for oseltamivir**	**Conclusion:** **oseltamivir**	**Mean IC50 (nM) for zanamivir**	**Fold change to wild type for zanamivir**	**Conclusion:** **zanamivir**
5: 3MDCK	H275Y	440	880	HRI	0.03	2.1	NI
6: 2MDCK	H275Y	245.8	491.6	HRI	0.03	2.13	NI
8: 3MDCK	H275Y	250.9	501.8	HRI	0.02	1.59	NI
A/California/07/2009	Wild type	0.5	Ref	NI	0.01	Ref	NI

HRI: highly reduced inhibition; IC50: 50% inhibitory concentration; MDCK: Madin-Darby canine kidney cells; NI: normal inhibition; ref: reference.

The mean IC50 is indicated in nM for both oseltamivir and zanamivir as well as the fold change to wild type control virus A/California/07/2019(H1N1pdm09).

## Discussion

This study describes a case of zanamivir and oseltamivir resistant influenza A(H1N1)pdm09 virus investigated in details with the use of NGS. Oseltamivir is the drug of choice when treating severely ill patients infected with influenza virus. Studies have shown that resistance towards oseltamivir develops fast within one week of treatment [1]. This coheres with the fact that only one mutation (H275Y) is needed to induce resistance against oseltamivir in H1N1pdm09 virus [21]. Antiviral resistance of influenza viruses against zanamivir is more rarely reported than oseltamivir resistance. The reason for this is likely due to the more extensive use of oseltamivir compared with zanamivir. H1N1pdm09 viruses with both I223R and H275Y mutations are shown to have increased resistance against both oseltamivir and zanamivir [22]. LeGoff et al. 2012 [1] shows that I223R alone confers reduced susceptibility to both oseltamivir and zanamivir and is primarily selected by oseltamivir. In this study we observed that the I223I/R mutation was selected after the introduction of zanamivir treatment, whereas the H275Y mutation was induced rapidly after initiation of oseltamivir treatment.

In immunocompromised patients the frequency of developing antiviral resistance against oseltamivir due to the H275Y mutation in connection to treatment can reach 13% [23] which is a substantially higher frequency compared with overall reporting of resistant H1N1pdm09 viruses which in the 2014/15 season was 0.4% [24]. Prolonged shedding of influenza virus in immunocompromised patients is well known and studies have provided evidence that the prolonged virus shedding can result in the emergence of additional mutations in the NA gene [1,25,26]. This indicates evolution of the viruses and the emergence of an increasingly more complex viral population in the antiviral-treated patient. This study contributes with further data to support this, as additional mutations were observed. The additional mutations discovered concerned amino acid substitutions both in the active and non-active site of the NA molecule, some of which have previously been described as involved in antiviral resistance, e.g. G147R and S247N [16,18]. In a recent published study by Takashita et al. [18] it was reported that H1N1pdm09 harbouring dual substitution at positions H275Y/G147R had a highly reduced inhibition by oseltamivir and peramivir, whereas, inhibition was within the normal range with zanamivir. The additional amino acid changing mutations not earlier described to induce antiviral resistance on their own, deserves further studies to clarify their potential effect on antiviral resistance. It could perhaps be an evolvement toward better fitness in the presence of the H275Y and I223R mutations under the selection of the antiviral drugs.

All samples were by default investigated by Sanger sequencing, however, due to the complex populations with mixed nt at important sites for antiviral resistance, NGS was performed to achieve deep sequencing and to provide the possibility to investigate minority variants. Interestingly, we found a larger proportion of minority variants by NGS. Furthermore, many of the mixed nt found by Sanger sequencing were confirmed by NGS which could additionally reveal the frequency of the different amino acid conferred by the nt variants. This underlines the limitations in interpretation of diverse viral populations/quasispecies using Sanger sequencing.

The present study emphasises the importance of rapid antiviral resistance testing during the course of a prolonged infection. The first sample submitted specifically for testing of antiviral resistance was obtained 96 days after the first influenza positive test. In the meantime the patient had been treated with two courses of oseltamivir and zanamivir treatment had been initiated. However, retrospective testing showed that the oseltamivir resistance mutation H275Y had been induced already after the first oseltamvir treatment, which means that the second course of oseltamivir treatment for 2 months had likely limited effect on the influenza virus infection. Whether an early antiviral resistance test revealing resistance towards oseltamivir and a subsequent rapid decision to shift to zanamivir could have improved the clearance of infection is however unknown. Studies indicate that immunocompromised patients have a major risk for prolonged shedding of influenza virus, and this in combination with the questionable effect of antivirals against influenza virus administered later than 48 hours after symptom onset, makes it difficult to predict the outcome of treatment [4,27]. Development of antiviral resistance in influenza virus in hospitalised patients poses a concern for nosocomial transmission, and a further risk of spreading mutant viruses. In particular, the prolonged infections frequently observed in immunocompromised patients can foster adaptation of increased fitness for viruses harbouring the antiviral resistance mutations. Close monitoring of these patients and of the development of antiviral resistance is necessary, and preventative measures against viral transmission both in the hospital setting and in the community need to be implemented.

Due to the limited amount of sample material it was not possible to investigate the phenotypic antiviral resistance characteristics in the NA inhibition assay. It is difficult to assess the importance of mixed mutations and the direct effect on antiviral resistance, without the opportunity to test in a phenotypic assay. From the clinical point of view, the patient’s condition did not improve with the administration of antivirals. Moreover laboratory testing revealed that the antiviral drugs had a limited effect against virus shedding, with emergence of resistance against both oseltamivir and zanamivir in the viral population infecting the patient. During cell propagation new mutations appeared which most likely can be attributed to cell adaptation, as the original samples did not contain the mutations but were represented in the virus isolates only. Cell adaptation of influenza viruses during propagation in MDCK cells is a normally observed phenomenon [28]. It is also problematic to perform phenotypic NA inhibition tests requiring virus isolation, when a sample contains a mixture of mutated resistant and wild type viruses. Indeed, during propagation there is a selection of viruses [26] and in this case the wild type virus was selected when propagation was performed without addition of antivirals to the growth medium. By addition of antivirals it was possible to rescue viruses with the combination of both I223R and H275Y mutations but none of the viruses displayed adequate NA activity to perform NA inhibition test. NA inhibition can be further assessed in a plaque reduction assay. However, due to limited amount of sample material left, this was not an option in our case. The lack of measurable NA activity could be due to the modifications of the NA protein caused by the induced mutations, even though it was possible for the virus to replicate in the cell cultures, or it could be due to remnants of antivirals in the cell growth medium interfering with the test.

An interesting finding in the study was the different antiviral resistance mutation profiles of two samples collected on the same day. The samples were obtained from a nasopharyngeal swab and a BAL, respectively, and the resistance mutation profiles differed, with the BAL sample having a higher frequency of the antiviral resistance mutations E119G, I223R and H275Y, whereas the nasopharyngeal sample, had a low frequency of additional mutations not found in the BAL sample. This could indicate a difference in the viral populations replicating in the upper and lower respiratory tract, respectively. This finding could be of importance when considering the sampling site for antiviral resistance testing, as the antiviral resistance profile for treatment evaluation could be misleading depending on the sampling site. Further studies on the compartmentalisation of influenza virus in the infected respiratory tract are needed.

## Conclusion

The rapidly evolving antiviral resistance observed in this case, emphasises the importance of timely antiviral resistance testing during treatment of influenza virus infection in order to change treatment regime and avoid unnecessary administration of ineffective medicaments, as well as preventing spread of antiviral resistant viruses.

Surveillance of antiviral susceptibility and research in the development of antiviral resistance in influenza virus is important to prevent the spread of antiviral resistant viruses, both in the hospital setting with risk group patients, and on a larger scale in the general population. The study contributes to the expansion of knowledge regarding the complexity of treating immunocompromised patients with antivirals, and the ecology of the influenza A(H1N1)pdm09 viral population under the selective pressure of antivirals. The study furthermore suggests that compartmentalisation of antiviral resistant viruses in the respiratory tract is of importance for considering the sampling site for antiviral resistance testing.
